# Supplementary figures and images for: Inferring bona fide Differentially Expressed Genes and Their Variants Associated with Vitamin K Deficiency Using a Systems Genetics Approach
Source: Genes (Basel). 2022 Nov 9;13(11):2078. doi: 10.3390/genes13112078 (PMC9690332; doi:10.3390/genes13112078)

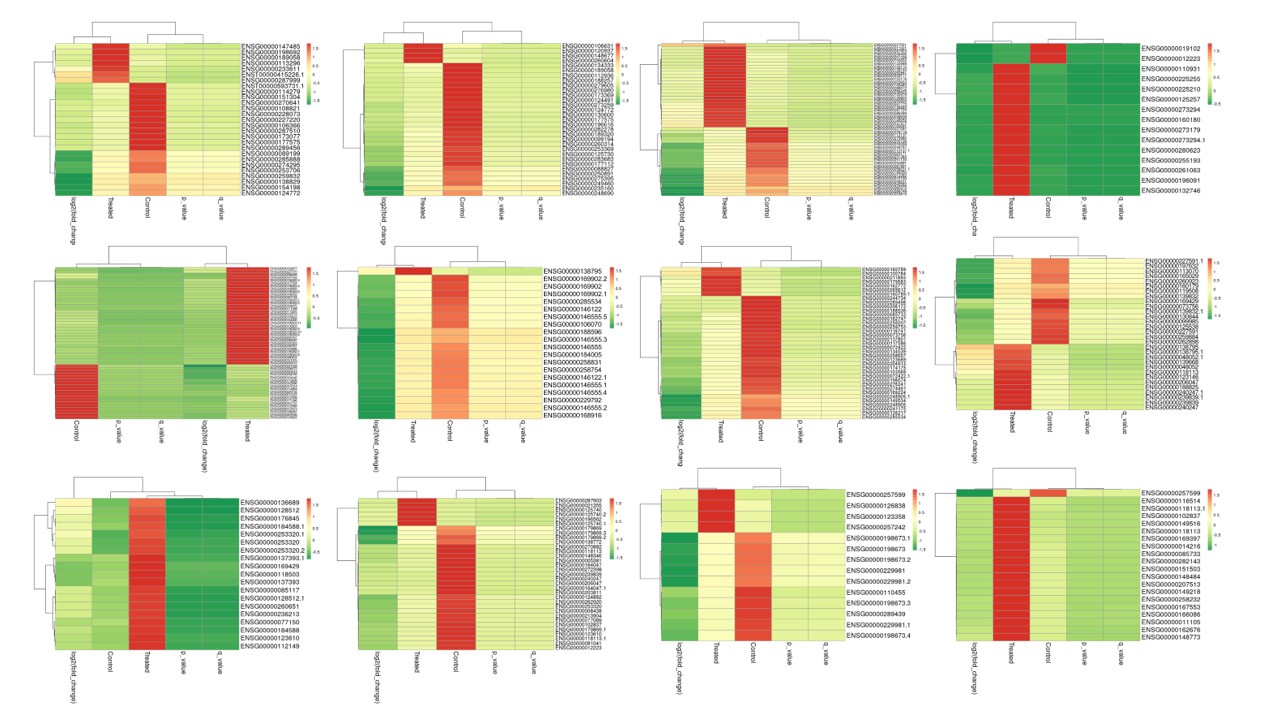

Supplement: Supplementary file 1 [file genes-13-02078-s001.zip › Supplementary Figure S1.jpg]

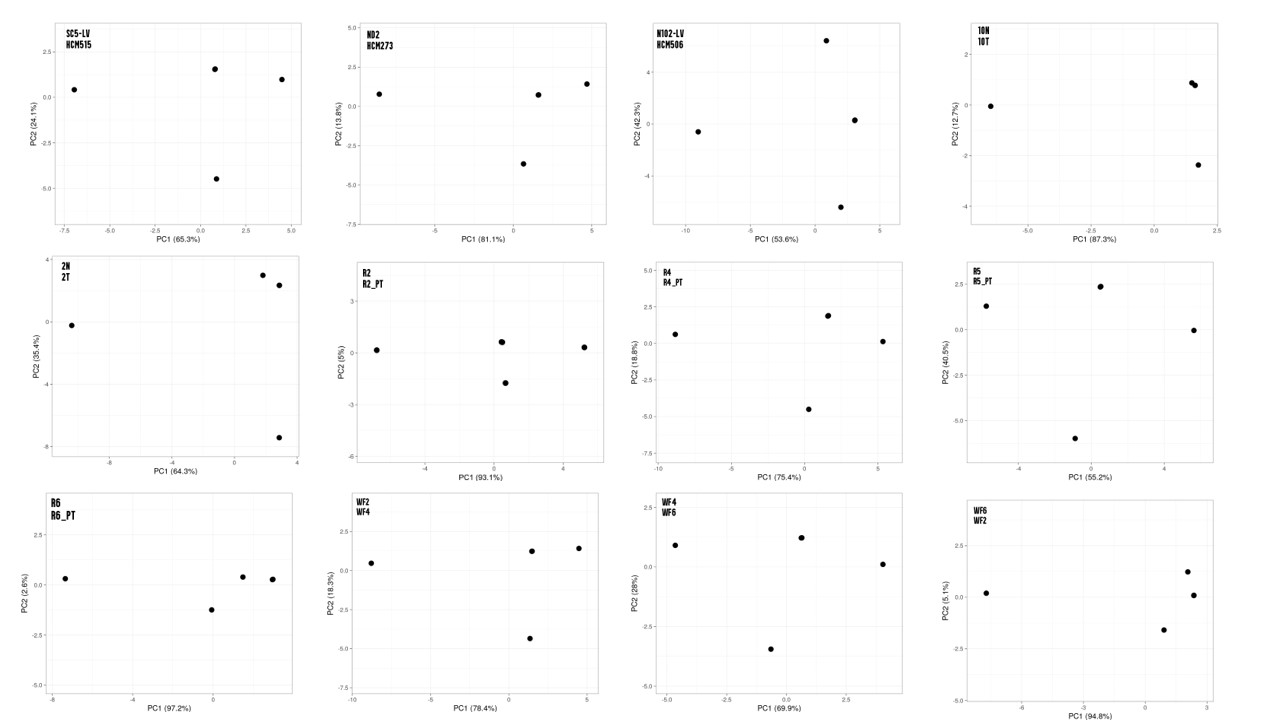

Supplement: Supplementary file 1 [file genes-13-02078-s001.zip › Supplementary Figure S2.jpg]
